# Downregulation of miR-335-5P in Amyotrophic Lateral Sclerosis Can Contribute to Neuronal Mitochondrial Dysfunction and Apoptosis

**DOI:** 10.1038/s41598-020-61246-1

**Published:** 2020-03-09

**Authors:** Noemi De Luna, Joana Turon-Sans, Elena Cortes-Vicente, Ana Carrasco-Rozas, Ignacio Illán-Gala, Oriol Dols-Icardo, Jordi Clarimón, Alberto Lleó, Eduard Gallardo, Isabel Illa, Ricardo Rojas-García

**Affiliations:** 1 Neuromuscular Diseases Laboratory - Biomedical Research Institute SantPau, Universitat Autónoma de Barcelona, Barcelona, Spain; 2 Department of Neurology, Neuromuscular Diseases Unit, Hospital de la Santa Creu i Sant Pau, Universitat Autònoma de Barcelona, Barcelona, Spain; 3 Centro de Investigación Biomédica en Red de Enfermedades Raras (CIBERER), Valencia, Spain; 4 Sant Pau Memory Unit, Department of Neurology, Hospital de la Santa Creu i Sant Pau, Biomedical Research Institute Sant Pau, Universitat Autònoma de Barcelona, Barcelona, Spain; 5 Centro de Investigación Biomédica en Red de Enfermedades Neurodegenerativas (CIBERNED), Valencia, Spain

**Keywords:** Neurological disorders, Neurodegenerative diseases, Neuromuscular disease

## Abstract

Amyotrophic lateral sclerosis (ALS) is a neurodegenerative disease for which the pathophysiological mechanisms of motor neuron loss are not precisely clarified. Environmental and epigenetic mechanisms such as microRNAs (miRNAs) could have a role in disease progression. We studied the expression pattern of miRNAs in ALS serum from 60 patients and 29 healthy controls. We also analyzed how deregulated miRNAs found in serum affected cellular pathways such as apoptosis, autophagy and mitochondrial physiology in SH-SY5Y cells. We found that miR-335-5p was downregulated in ALS serum. SH-SY5Y cells were transfected with a specific inhibitor of miR-335-5p and showed abnormal mitochondrial morphology, with an increment of reactive species of oxygen and superoxide dismutase activity. Pro-apoptotic caspases-3 and 7 also showed an increased activity in transfected cells. The downregulation of miR-335-5p, which has an effect on mitophagy, autophagy and apoptosis in SH-SY5Y neuronal cells could have a role in the motor neuron loss observed in ALS.

## Introduction

Amyotrophic lateral sclerosis (ALS) is a progressive neurodegenerative disease. It is classically characterized by weakness and atrophy due to loss of lower motor neurons in the brainstem and spinal cord and loss of upper motor neurons in the motor cortex^1^. Degeneration, however, is now considered to be more widespread and not restricted to the motor system. Up to 50% of patients show evidence of behavioral and cognitive dysfunction, suggesting pathologic changes involving the frontal and temporal cortex, and sometimes fulfilling diagnostic criteria of frontotemporal dementia (FTD). These disorders are now considered to be manifestations within the clinicopathological spectrum of an underlying mechanism of neurodegeneration^2,3^.

The molecular pathogenic mechanism of ALS onset and progression is not completely known. Environmental factors have been described as potential contributors to neurodegeneration and ALS progression, but none have proven to be causative^4,5^. Mutations in more than 25 genes have been implicated in familial (fALS) and sporadic ALS (sALS) forms^6^, suggesting a wide heterogeneity in the molecular bases of the disease. Several ALS-causing genes encode proteins involved in RNA metabolism processes, such as RNA transcription, splicing, mRNA transport and microRNAs (miRNAs) biogenesis^7^. Dysfunction of these mechanisms is not exclusive and may lead to diverse cellular abnormalities. Epigenetic mechanisms, such as miRNAs, could regulate the expression of genes involved in common cellular pathways that are disrupted in ALS and therefore have an impact on ALS phenotype^8^. MiRNAs are small, non-coding RNA molecules of approximately 22 nucleotides that regulate gene expression in fundamental cellular processes and, post-transcriptionally, in the translation levels of target mRNA transcripts^9^. A single miRNA can regulate multiple transcripts and a single transcript can be regulated by multiple miRNAs. MiRNAs mediate silencing of target genes guided by Argonaute proteins, forming the silencing complex which promotes translational repression and degradation of targeted mRNA^10^. Numerous miRNAs are also temporally and spatially regulated with transcription factors or epigenetic mechanisms^11^. Deregulation of miRNA function is associated with numerous diseases^12^, including ALS^13,14^. Therefore, profiling of the miRNA expression could serve as a potential tool for ALS diagnosis, prognosis, and follow up, or to gain insight into the pathophysiology of the disease^13^.

Oxidative stress and mitochondrial damage are key features of most neurodegenerative diseases^15,16^, including ALS^17^, suggesting that mitochondrial dysfunction might contribute to motor neuron pathology^18,19^. Mitochondrial oxidative stress is also responsible for mitochondrial DNA damage in spinal motor neurons of sporadic ALS patients^20^. Mitochondrial dynamics play an important role in the neuronal survival by maintaining ATP biogenesis, Ca^2+^ homeostasis and regulating oxidative stress. Mitochondria produce ATP through the OXPHOS system, and at the same time, they generate reactive species of oxygen (ROS)^21,22^. When ROS production is excessive it causes oxidative stress in the cell^23^. Mitochondrial components may be damaged by ROS and can cause dysfunction of respiratory enzyme activity and mitochondrial depolarization^24^. Oxidative stress also induces cell death, releasing pro-apoptotic proteins^25^. A central role for autophagy in ALS is well supported by the pathology of the disease, which commonly includes the accumulation of protein aggregates and swollen mitochondria in motor neurons of affected patients^26^. In this study, we aimed to analyze the miRNA expression pattern in ALS serum and test the hypothesis that deregulation of some miRNA affects the mitochondrial physiology of neuronal cells.

## Results

### In the discovery phase thirteen miRNA had deregulated expression in ALS serum compared to controls

**Figure 1:**
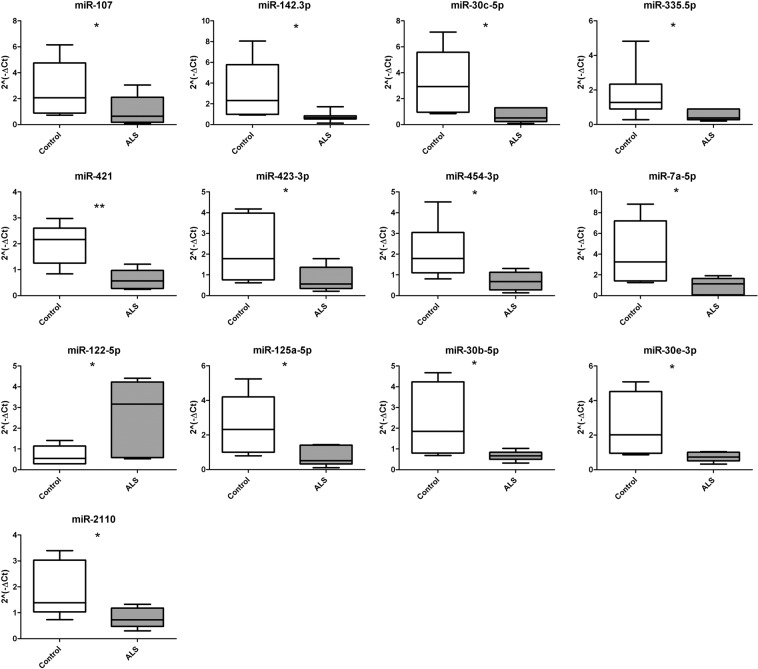
Deregulated miRNA expression in the discovery phase. Normalized relative expression levels of serum miRNAs in ALS patients compared to healthy controls. Data are presented as mean ± SD. *p ≤ 0.05 (Mann-Whitney U test). Graphics of miRNA expression were created using GraphPad Prism, version 5.03 (https://www.graphpad.com/scientific-software/prism/).

Seven ALS patients and six healthy controls were analyzed in the discovery phase. The clinical and demographic characteristics of ALS patients are included in Table 1. We observed significantly different expression levels between ALS patients and controls in 13 out of the 185 mRNAs included in the panel from the discovery phase (Fig. 1). The 13 deregulated miRNAs were: miR-107, miR-142-3p, miR-30c-5p, miR-335-5p, miR-421, miR-423-3p, miR-454-3p, miR-7a-5p, miR-122-5p, miR-125a-5p, miR-30b-5p, miR-30e-5p and miR-2110.

**Table 1:** Demographic and clinical characteristics of patients included in the miRNAs study.

Id	Gender	Age	Age at onset	Age at death	Mutated gene	Mutation	Phenotype	Onset	Familial
**1**	**F**	**69,8**	**67,6**	**71,1**	**—**	**—**	**ALS**	**espinal**	**No**
**2**	**M**	**64,6**	**62,2**	**67,7**	**—**	**—**	**ALS**	**espinal**	**No**
**3**	**F**	**70,3**	**68,6**	**Alive**	**—**	**—**	**ALS**	**espinal**	**No**
**4**	**M**	**68,4**	**60,9**	**Alive**	**—**	**—**	**ALS**	**espinal**	**No**
**5**	**F**	**49,4**	**46,0**	**Alive**	**SOD1**	**p.Asp77Val**	**fALS**	**espinal**	**Yes**
**6**	**M**	**39,9**	**34,3**	**Alive**	**TBK1**	**p.Gly121Asp**	**ALS**	**espinal**	**No**
**7**	**M**	**60,9**	**60,5**	**Alive**	**—**	**—**	**ALS**	**espinal**	**No**
8	M	57,0	55,5	Alive	—	—	ALS	espinal	No
9	M	78,4	77,8	78,9	—	—	ALS	espinal	No
10	M	72,2	64,0	72,7	—	—	ALS	espinal	No
11	F	71,0	70,1	72,5	—	—	ALS	bulbar	No
12	M	75,0	74,4	75,2	—	—	ALS	espinal	No
13	M	61,9	53,9	64,2	—	—	ALS	espinal	No
14	M	52,7	52,0	Alive	—	—	ALS	espinal	No
15	M	71,5	69,0	Alive	—	—	ALS	bulbar	No
16	F	78,3	73,9	Alive	—	—	ALS-FTD	bulbar	No
17	M	64,2	35,3	66,6	SOD1	p.GLy38Arg	fALS	espinal	Yes
18	F	71,9	68,5	Alive	—	—	ALS	espinal	No
19	F	64,4	62,8	65,5	—	—	ALS	bulbar	No
20	F	56,6	54,3	57,3	—	—	ALS	espinal	No
21	F	32,1	25,7	Alive	—	—	ALS	espinal	No
22	F	55,9	42,0	57,6	SOD1	p.Ile113Met	fALS	espinal	Yes
23	F	85,7	84,6	86,2	—	—	ALS	bulbar	No
24	M	68,0	67,4	71,6	—	—	ALS	espinal	Yes
25	M	55,6	54,0	57,8	—	—	ALS-FTD	bulbar	Yes
26	M	46,4	45,1	47,5	FUS	p.Arg521His	fALS	espinal	Yes
27	M	65,1	62,2	65,5	TARDBP	p.Arg90Val	ALS-FTD	bulbar	Yes
28	F	86,3	84,2	86,3	—	—	ALS	bulbar	No
29	M	82,9	75,3	Alive	—	—	ALS	espinal	No
30	F	69,2	65,2	70,2	—	—	ALS	espinal	No
31	F	66,6	63,4	67,1	—	—	ALS	espinal	No
32	F	53,5	38,5	Alive	SOD1	p.Gly37Arg	fALS	espinal	Yes
33	F	62,2	57,3	64,9	—	—	ALS	espinal	No
34	M	64,5	61,9	64,7	—	—	ALS	espinal	No
35	F	74,1	64,7	Alive	—	—	ALS	espinal	No
36	F	63,3	60,7	Alive	—	—	ALS	espinal	No
37	F	73,1	70,4	74,0	—	—	ALS-FTD	bulbar	No
38	F	82,9	81,9	83,2	—	—	ALS	bulbar	No
39	M	79,6	71,6	80,5	—	—	ALS	espinal	No
40	M	73,7	73,3	73,8	—	—	ALS	bulbar	No
41	F	69,3	68,0	70,9	—	—	ALS	espinal	No
42	F	61,8	59,5	60,5	—	—	ALS	espinal	No
43	F	72,5	70,3	73,4	—	—	ALS	bulbar	No
44	M	46,0	42,6	47,7	—	—	ALS	bulbar	No
45	F	71,1	70,7	71,2	—	—	ALS	espinal	No
46	F	68,9	68,4	71,2	—	—	ALS	bulbar	No
47	F	30,8	29,8	Alive	FUS	p.Ser57del	fALS	espinal	Yes
48	F	56,4	54,6	Alive	—	—	ALS	espinal	No
49	F	66,4	66,4	67,1	—	—	ALS	espinal	No
50	M	70,1	68,0	70,6	—	—	ALS-FTD	bullbar	No
51	M	50,9	50,1	52,4	—	—	ALS	bulbar	No
52	M	75,0	74,0	Alive	—	—	ALS	espinal	No
53	F	63,1	62,3	53,8	—	—	ALS-FTD	espinal	No
54	F	63,8	62,8	65,5	—	—	ALS	bulbar	No
55	M	76,4	75,5	77,2	—	—	ALS	bulbar	No
56	M	60,1	58,7	62,3	—	—	ALS	bulbar	No
57	M	74,0	67,5	Alive	—	—	ALS	espinal	No
58	F	79,0	78,0	79,0	—	—	ALS	espinal	No
59	H	62,9	61,9	64,7	—	—	ALS	espinal	No
60	F	69,7	67,6	71,1	—	—	ALS	espinal	No

Sera from patients 1 to 7 (in bold) were used in the discovery phase to study miRNAs profile. Sera from patients 8 to 60 were analyzed in the validation phase.

### miR-335-5p is differentially expressed between ALS serum and controls in the validation phase

**Figure 2:**
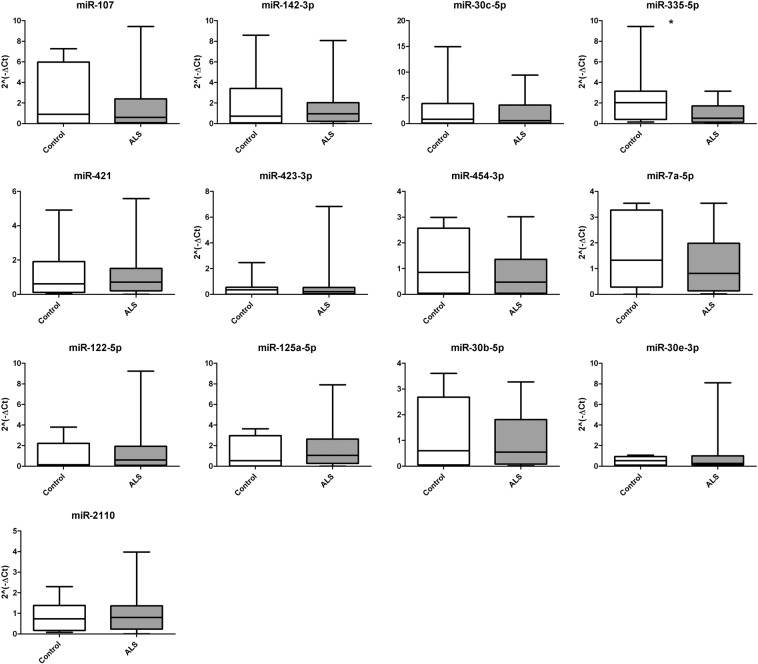
MiRNA expression in validation phase. Only miR-335-5p expression showed statistically significant differences between ALS patients and healthy controls. Normalized relative expression levels of serum miRNAs in ALS patients compared to healthy controls. Data are presented as mean ± SD. *p ≤ 0.05 (Mann-Whitney U test). Graphics of miRNA expression were created using GraphPad Prism, version 5.03 (https://www.graphpad.com/scientific-software/prism/).

In the validation phase we assessed the expression of the 13 miRNA deregulated in the discovery phase in sera from 53 ALS patients and 23 healthy controls. After Gene-Globe analysis (Qiagen, Hilden, Germany), the expression of miR-335-5p in serum showed a significant reduction (−1.67 fold decrease, p = 0.023) in ALS patients compared to controls. The Mann-Whitney U test of each miRNA expression between ALS and controls showed statistically significant differences in miR-335-5p (Fig. 2) No significant differences were observed in the other miRNAs analyzed in the validation phase (Fig. 2).

### Inhibition of miR-335-5p altered mitochondrial dynamics

To assess the involvement of decreased miR-335-5p in mitochondrial dynamics, SH-SY5Y cells were transfected with an antisense miR-335 vector, a negative control (NC) together with mKeima-Red-Mito7. The fluorescent protein Keima has an excitation spectrum that changes according to pH. In a neutral environment the protein is excited at a short wavelength and displays a green signal, whereas in an acidic environment it is excited at a long wavelength and displays a red signal^27^. The miR-335 inhibitor forms a duplex with the miRNA guide strand and prevents miRNA from binding to its intended target. At 24 h after miR-335 inhibitor transfection, miR-335-5p levels were drastically reduced (Ct ≥ 40) while the NC showed a Ct of 32.2 ± 0.5.

**Figure 3:**
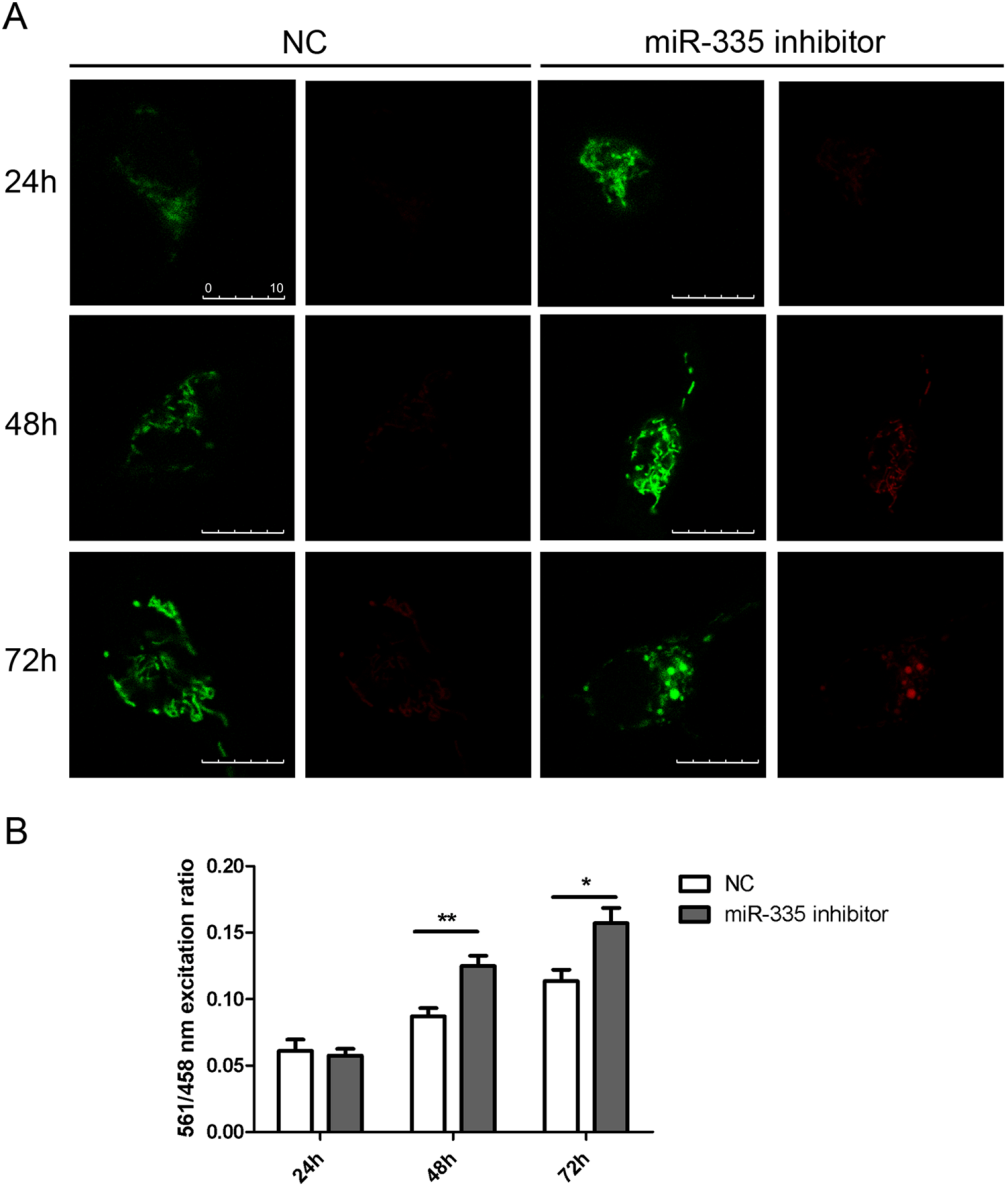
Co-transfection of miR-335 inhibitor or NC together with Keima Red. (**A**) Confocal images showed an increased pool of mitochondria 48 and 72 h after transfection with the miR-335 inhibitor.0 (**B**) 561/458 nm excitation ratio showed an increase number of red mitochondria (excited at 561), indicating there localization in an acidic environment. The graphic representation was created using GraphPad Prism, version 5.03, (https://www.graphpad.com/scientific-software/prism/).

After 24 h of transfection, mitochondria displayed no morphological changes. However, 48 h and 72 h after transfection, the number of mitochondria displaying a red signal increased significantly, indicating that they were located in acidic environment, probably due to mitophagy mediated by lysosomes (Fig. 3A). Furthermore, at 72 hours some cells presented round-shaped mitochondria which differed from those in NC. Excitation at 561 nm showed red mitochondria located in an acidic environment whereas excitation at 458 nm showed green mitochondria under a neutral environment. The excitation ratio 561/458 nm thus shows the ratio between mitophagic and healthy mitochondria. This ratio showed statistically significant differences at 48 h (**p < 0.01) and 72 h (*p < 0.05) between the miR-335 inhibitor and NC transfected cells (Fig. 3B).

**Figure 4:**
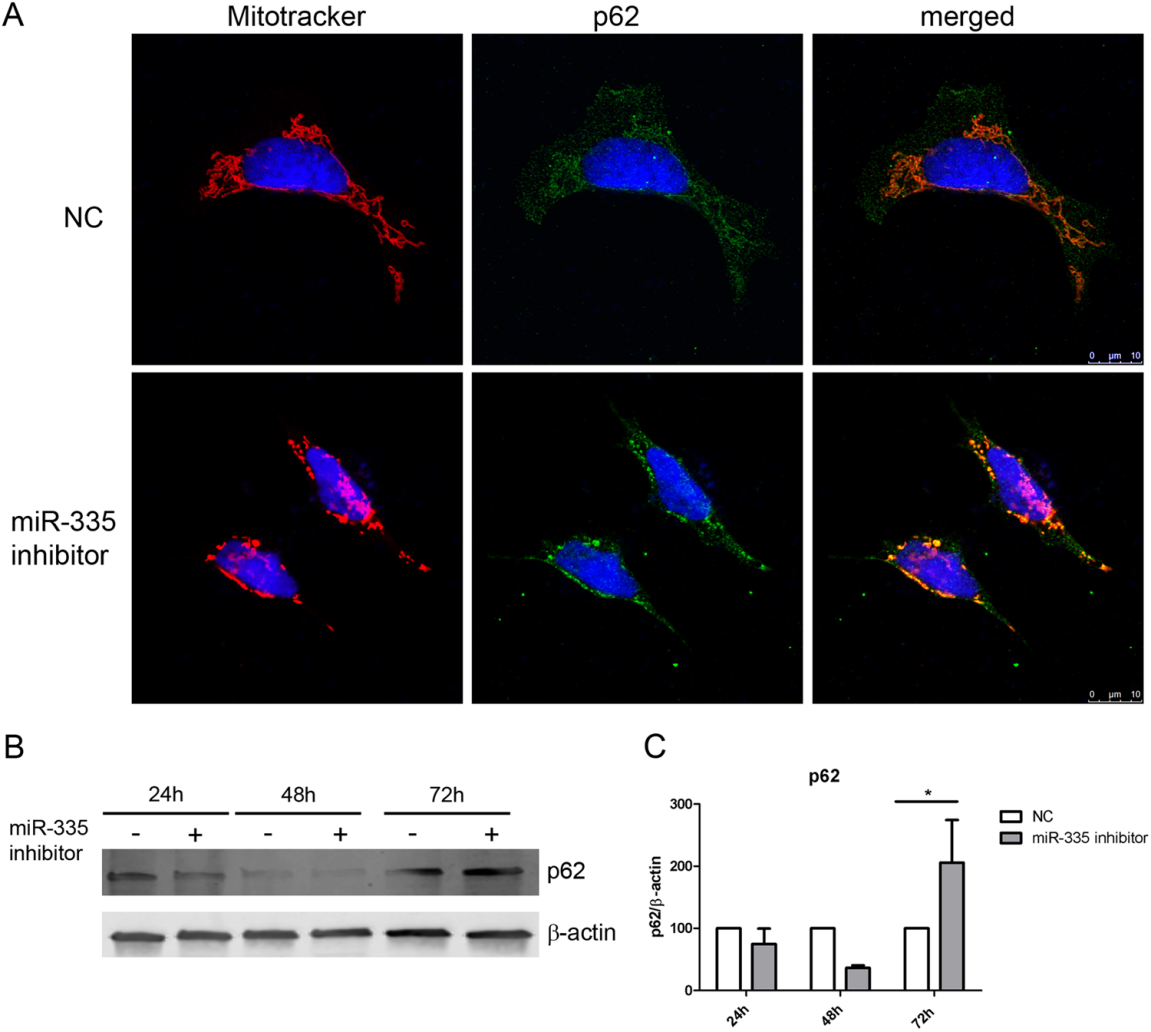
Mitochondria co-localize with p62 after miR-335 inhibitor transfection. (**A**) Co-localization of mitochondria (red) with p62 autophagic marker (green), 72 h after transfection with miR-335 inhibitor. (**B**) Representative WB of p62 in cells after 24, 48 or 72 hours after transfection. Bands corresponding to p62 and β-actin were cropped from the same gel. (**C**) WB quantification showed a decrease in p62 expression at 48 h, and an increase at 72 h after transfection, indicating potential impairment of autophagy in transfected cells. The graphic representation was created using GraphPad Prism, version 5.03, (https://www.graphpad.com/scientific-software/prism/).

At 72 h after transfection, some of the miR-335 inhibitor transfected cells presented swollen and rounded mitochondria, and they co-localized with p62, an autophagosome marker. In miR-335 inhibitor transfected cells, p62 remained accumulated on perinuclear clustered-mitochondria, while in NC no p62 positive vacuoles were observed (Fig. 4A). The Western-Blot (WB) of p62, showed an increased expression (*p < 0.05) at 72 h (Fig. 4B,C), indicating a potentially abnormal autophagy process in SH-SY5Y cells after miR-335 inhibitor transfection.

### Oxidative stress is increased in miR-335 inhibitor transfected cells

**Figure 5:**
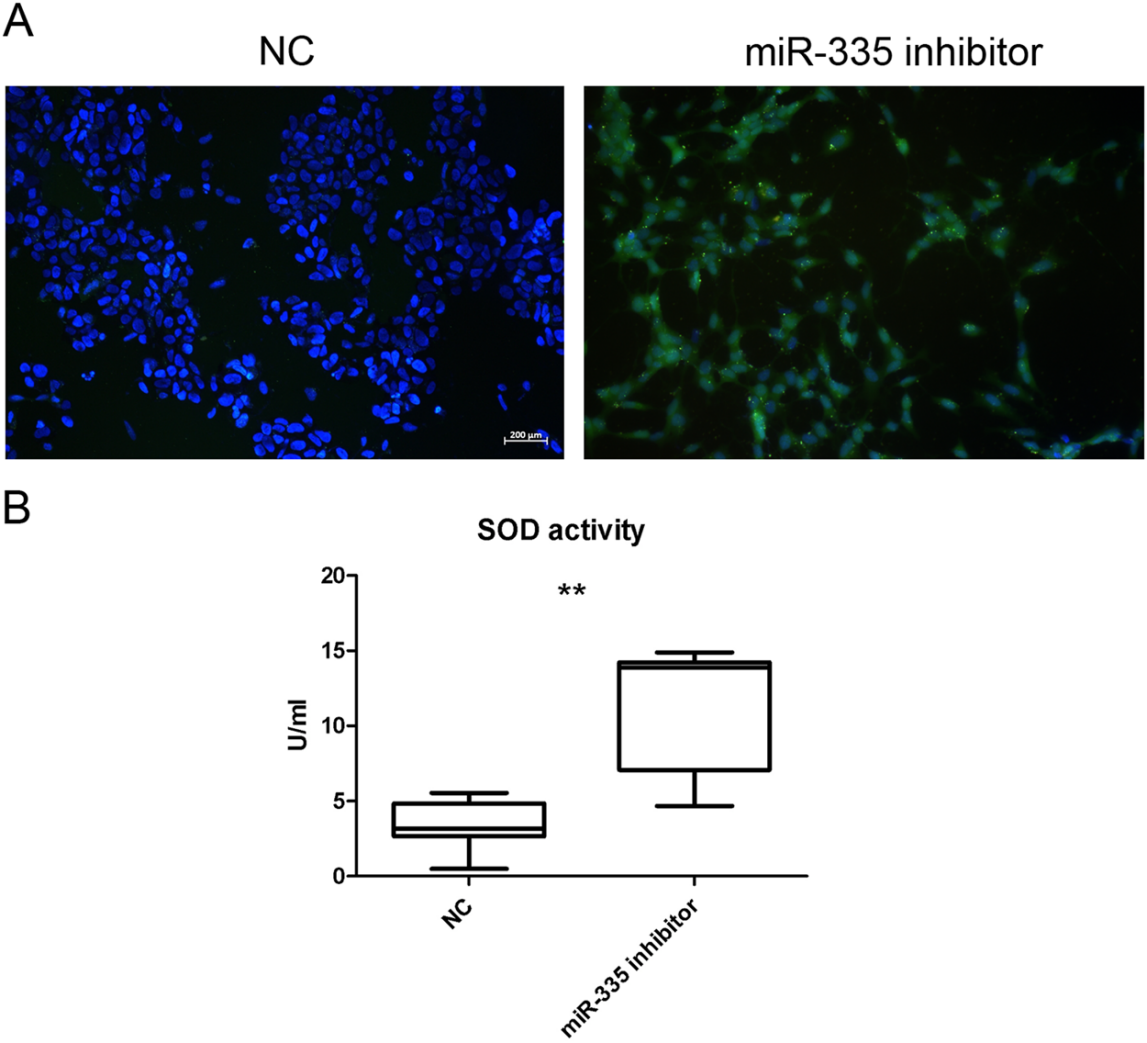
Increment of ROS and SOD activity after miR-335 inhibitor transfection. (**A**) ROS detection in SH-SY5Y transfected cells 24 h after transfection correlated with an increment of SOD activity as a measure to regulate ROS levels (**B**). The graphic representation was created using GraphPad Prism, version 5.03, (https://www.graphpad.com/scientific-software/prism/).

The generation of ROS in cells has a deleterious effect on mitochondria normal function. For this reason, we studied the production of ROS in SH-SY5Y cells transfected with the miR-355 inhibitor. Cells transfected with this inhibitor showed green fluorescence, indicating that the fluorogenic probe of the assay was oxidized by ROS. This was not observed in NC (Fig. 5A). We also observed a significant increase in SOD activity 24 h after miR-335 inhibitor transfection (t-test **p < 0.01) compared to NC (Fig. 5B). No differences were observed at 48 and 72 h after transfection (Data not shown).

### Inhibition of miR-335-5p activates caspase 3/7 apoptotic pathway

**Figure 6:**
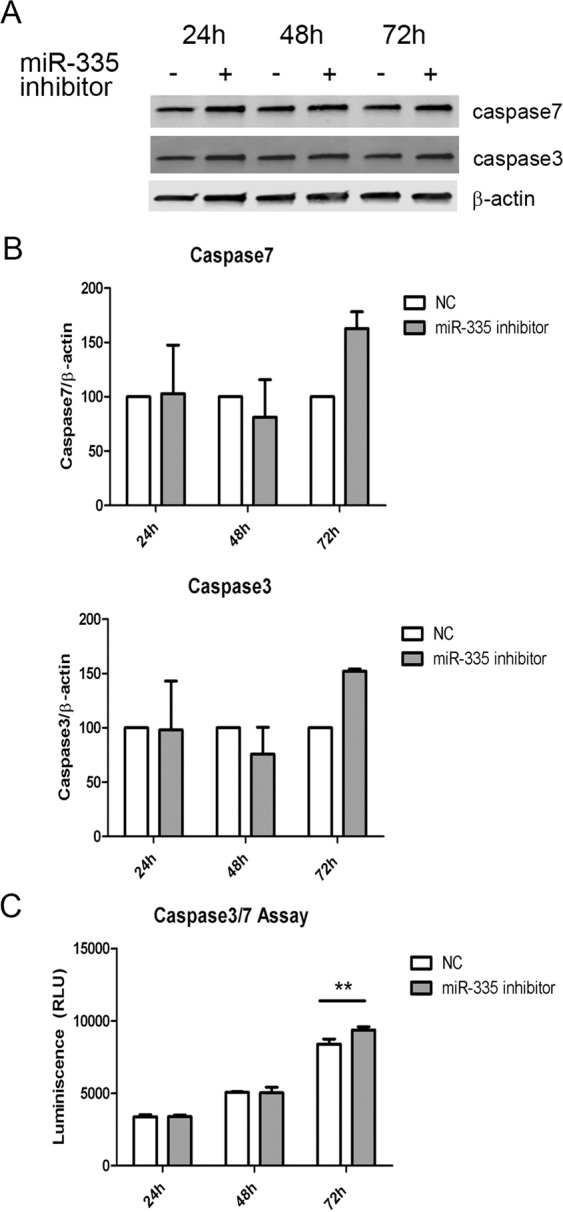
Abnormal activity of apoptosis effector caspases after miR-335 inhibitor transfection. (**A**) Representative WB of caspase-3 and 7 expressions after miR-335 transfection. Bands corresponding to caspase-7, caspase-3 and β-actin were cropped from the same gel. We can quantify the bands corresponding to caspase-7 and caspase-3 (B), and although no significant differences were observed, when we measured the activity of these caspases, we observed a significant increase in caspase activity (**p < 0.01) (**C**). The graphic representations were created using GraphPad Prism, version 5.03, (https://www.graphpad.com/scientific-software/prism/).

To determine whether apoptosis could be influenced by miR-335-5p downregulation, we analyzed the expression of caspase-3 and caspase-7 as final effectors of the classical apoptotic pathway. Furthermore, *CASP-7* is directly targeted by miR-335-5p (http://www.targetscan.org). After cell transfection with the miR-335 inhibitor, we observed a 1.63 fold-increase in caspase-7 and a 1.52 fold-increase of expression in caspase-3 at 72 h. However, these differences did not reach statistical significance (Fig. 6A,B).

To study whether the increased expression observed by WB would correlate with an increment of caspase 3/7 activity, we measured this by luminescence ((RLU = Relative Light Units)). No differences were observed at 24 h or 48 h after transfection. However, a significant increase of caspase 3/7 activity was observed at 72 h (2way ANOVA, p < 0.01)(Fig. 6C).

## Discussion

Our screening of 185 miRNAs in serum from ALS patients and controls revealed a downregulation of miR-335-5p in ALS patients, and this was replicated in an independent validation cohort. MiR-335-5p has high potential activity since it targets 2544 genes^28^. Changes in the expression of this miRNA could therefore have an impact on multiple cell functions.

In other motor neuron diseases, such as spinal muscle atrophy, miR-335-5p was highly reduced in neural stem cells^29,30^. Our results in ALS patients add evidence to a possible role of this specific non-coding RNA in the pathogenic process of motor neuron degeneration. It has been reported that miR335-5p downregulation is necessary to stabilize plasticity and memory in mice^31^ and may play a role in the appearance of cognitive symptoms in ALS patients with concomitant FTD, thus theoretically, contributing to the whole clinical spectrum of the disease.

Previous studies have described that miR-206, miR-143-3p and miR-374b-5p are deregulated in ALS patients’ serum^32^. Our panel did not include miR-206 and we could not therefore test its expression in our samples. We did not observe significant differences in miR-143 expression in our panel. Although miR-374b-5p was represented in our panel and showed a tendency to be downregulated in ALS patients, no significant differences were reached between groups.

Low levels of miR-30b-5p and high expression of miR-2110 have been reported to correlate with more rapid progression of the disease^33^. Although we observed that levels of miR-30b-5p and miR-2110 were low in the serum of our ALS cohort, again our results did not reach statistical significance.

Our results suggest that downregulation of miR-335-5p affects mitochondrial dynamics, autophagy and apoptosis in SH-SY5Y neuronal cells. Mitophagy is the selective degradation of damaged mitochondria by autophagy, contributing to maintenance of a healthy population of mitochondria. Since damaged mitochondria lead to a collapse of cell homeostasis, mitophagy is believed to protect against diseases related to mitochondrial dysfunction, such as neurodegenerative disorders^34^. In our study, we observed a co-localization of mitochondria with p62. P62 can act as an autophagy receptor that connects autophagic substrates with autophagosomes^35^, and it is increased following induction of mitophagy^36^. These findings, together with the observation of mitochondria in an acidic environment, probably inside autophagic vacuoles, indicate that downregulation of miR-335-5p could induce mitophagy in SH-SY5Y cells. Oxidative stress and mitochondrial alterations are also observed in the muscle of SOD1mutant mice^37,38^. Under oxidative stress conditions, ROS production is dramatically increased, resulting in subsequent alteration of membrane lipids, proteins and nucleic acids^39^ which could induce cell death. We observed an increase of ROS in miR-335 inhibitor transfected cells and a proportional increase of SOD activity, suggesting an attempt to regulate ROS levels after miR-335-5p downregulation. Mitochondrial SOD2 and SOD1 are usually sufficient to remove superoxide efficiently to avoid propagation of damage in the other compartments of the cell^20^. Mitochondria regulate apoptosis in the nervous system both in healthy and pathological conditions^18^. Caspase-7 together with caspase-3 are executioner caspases that lead to apoptosis by cleaving specific sets of substrates^40^. Mature caspase-3 and 7 activation results in nuclear condensation and genomic DNA break up, among other cellular events. Since *CASP-7* is a direct target of miR-335-p, our results suggest that the downregulation of miR-335-p increases caspase-7 expression which in turn activates apoptosis and leads to cell death of SH-SY5Y neuronal cells. In fact, it has been reported that increased caspase-7 expression correlates with excessive neuronal cell death in some neurodegenerative disorders^41^. Therefore, caspase-7 activation constitutes a potential therapeutic target that could be beneficial in diseases such as ALS.

Our results provide evidence that downregulation of miR-335-5p may enhance mitophagy and apoptosis and support the notion that dysregulation of miRNAs may be involved in the pathogenic process of neuron degeneration in ALS.

## Patients and Methods

### Subjects, sample collection and isolation of miRNAs in serum samples

In this study, we analyzed samples from 89 subjects (60 ALS patients and 29 sex and age-matched healthy controls). The study was designed in two steps: a discovery phase (7 ALS patients and 6 healthy controls) and a validation phase (53 ALS patients and 23 healthy controls) (Table 1). ALS patients were prospectively recruited from the Motor Neuron Disease Clinic at Hospital de la Santa Creu i Sant Pau. They all fulfilled El Escorial revised criteria for probable, probable laboratory-supported, or definite ALS^42^ and underwent comprehensive cognitive and behavioral screening at the Sant Pau Memory Unit. Patients were categorized as ALS-FTD according to current diagnostic criteria for the behavioral variant of frontotemporal dementia^43,44^. Healthy controls were recruited from non-genetically related relatives and serum samples were taken after signed informed consent was obtained. All patients were able to sign an informed consent form by themselves. The study was approved by the local ethics committee (Ethic Committee of Investigation with Drugs from Fundación de Gestió Sanitària de l’Hospital de la Santa Creu i Sant Pau) in compliance with the 1964 Helsinki Declaration and its later amendments.

### Analysis of miRNA expression in serum

After whole blood samples were collected and left for 30 minutes to clot. The tubes were then centrifuged at 1800 rpm for 10 minutes. miRNA extraction was performed as previously described^45^.miRNAs were isolated from serum using the miRCURY RNA Isolation Kit – Biofluids (Qiagen). 200 µl of serum were lysed with lysis solution following manufacturer’s instructions. MiRNAs were isolated after isopropanol precipitation and centrifugation in spin columns. MS2 RNA (Roche, Basel, Switzerland) was added to the samples as a carrier to improve the efficiency of RNA isolation. 2 μl of isolated miRNAs were then retrotranscribed into a cDNA with the miRCURY LNA Universal RT microRNA PCR (Qiagen). A 1/60 cDNA dilution was used to perform each assay included in Serum/Plasma Focus microRNA PCR 384 wells Panels, (V4.M) (Qiagen) and to perform individual assays of the validation phase.

To ensure the quality of the results the samples used in the panels were selected according to two criteria: the efficiency of miRNA isolation and the level of haemolysis. We analyzed miR-451a and miR-23a-3p as haemolysis markers.

The study was designed in two steps: a discovery phase and a validation phase.

In the first step or discovery phase, we analyzed expression levels of 185 miRNA included in a PCR panel in 7 ALS patients (Table 1, in bold) and 6 age-matched healthy controls, to identify differentially expressed candidate miRNAs.

In the validation phase, the significantly deregulated miRNAs were analyzed in a larger cohort of patients and controls (53 ALS patients and 23 healthy controls).

The results of miRNAs expression were evaluated using Applied Biosystems SDS 2.4 software and the results were transformed into an Excel file. Mean and SD were calculated and statistical analysis was performed using Gene-Globe Data Analysis Center, an online platform provided by Qiagen. The p values were calculated based on a Student’s t-test of the replicate 2^(-ΔCt) values for each gene in the control group and in the ALS group. Samples were normalized using the global Ct mean of expressed miRNAs. Whether an miRNA was expressed or not was determined by the lower limit of detection that was set up to Ct 37.

### Discovery phase

The miRNAs profile was studied by qPCR using Serum/Plasma Focus microRNA PCR 384 wells Panels, (V4.M) (Qiagen). This tool includes 185 different miRNA oligonucleotides, all of which were analyzed in each patient (n = 7) and healthy control (n = 6).

The deregulated miRNAs between ALS patients and controls found in the discovery phase were assessed in a second step or validation phase, which included 53 ALS patients and 23 healthy controls (Table 1).

The results were analyzed using software developed by Anaxomics (Barcelona, Spain).

### Validation phase

For the validation phase, the 13 deregulated miRNAs identified in the discovery phase were evaluated in serum samples from 23 healthy controls and 53 ALS patients. miRCURY LNA miRNAs PCR Assays (Qiagen) were used for each of the miRNAs, and amplified fragments were detected using ExiLENT SYBR Green master mix (Qiagen). In total, we studied 13 candidate miRNAs (miR-107, miR-142-3p, miR-30c-5p, miR-335-5p, miR-421, miR-423-3p, miR-454-3p, miR-7a-5p, miR-122-5p, miR-125a-5p, miR-30b-5p, miR-30e-5p, miR-2110), 2 haemolysis controls (miR-451a and miR-23a-3p), and 4 exogenous controls (spike-ins UniSp2, UniSp4, UniSp5 and UniSp6), as described above.

### SH-SY5Y cell culture

Human neuroblastoma cell line SH-SY5Y was obtained from the general collection of the European Collection of Authenticated Cell Culture (ECACC n°9403034) and purchased at Sigma-Aldrich (St. Louis, MI, USA). SH-SY5Y cells were seeded on culture well plates which were coated with 0.15% gelatin (Sigma-Aldrich) for 30 minutes. SH-SY5Y were grown in proliferation media (EMEM (Lonza Group Ltd, Basel, Switzerland):F12 medium (Sigma-Aldrich) (1:1) supplemented with 15% FBS (Lonza), 2 mM glutamine (Lonza), 1 mM sodium pyruvate (Lonza) and penicillin-streptomycin (Lonza).

### SH-SY5Y transient transfection

For transfection experiments, SH-SY5Y cells were seeded at 20,000 cells/cm^2^.

We transfected 10 nM of IDT miRNA Inhibitor (Integrated DNA Technologies (IDT), Newark, NJ, USA), specific for miR-335-5p (miR-335 inhibitor). This oligonucleotide is perfectly complementary to the mature miRNA target. As a negative control (NC) we used a validated sequence (5′-ucguuaaucggcuauaauacgc-3′) developed by IDT.

To test mitophagy, we co-transfected the miRNA-335 inhibitor together with mKeima-Red-Mito-7 (a gift from Michael Davidson (Addgene plasmid # 56018; http://n2t.net/addgene:56018; RRID:Addgene_56018)). As a negative control, we co-transfected NC vector with mKeima-Red-Mito-7. MKeima-Red-Mito-7 contains a mitochondrial targeting sequence corresponding to the subunit VIII of human cytochrome C oxidase. The transfection reagent used was Fugene HD (Promega, Madison, WI, USA). The fluorescence was measured with Zeiss Zen software (Zeiss, Oberkochen, Germany), and the ratio 561/458 nm excited Keima fluorescence was calculated to reflect the underlying level of mitophagy. Images were acquired with a Leica Confocal microscope (Leica, Wetxlar, Germany).

### Mitochondrial staining and Immunohistochemistry

To study mitochondrial morphology we stained transfected cells with MitoTracker Red CMXRos (Thermo Fisher Scientific), a red-fluorescent dye that stains mitochondria in living cells.

After mitotracker incubation, transfected cells were permeabilized with blocking goat solution 5% together with Triton X-100 0.1% (Sigma-Aldrich) during 30 minutes. Cells were incubated with p62 antibody (1/200 dilution) (Enzo LifeSciences, NY, USA, Cat# BML-PW9860, RRID:AB_2196009) and goat-anti-rabbit Alexa 488 (1/500 dilution) (Molecular Probes, Eugene, OR, USA, Cat# A-11008, RRID:AB_143165) was used as secondary antibody. Images were acquired with a Leica Confocal microscope (Leica).

### Oxidative stress detection

To measure oxidative stress in transfected cells we used CellROX Green Reagent (Invitrogen, Carlsbad, CA, USA) following the manufacturer’s instructions. The cell-permeant dye is weakly fluorescent while in a reduced state but exhibits bright green photostable fluorescence upon oxidation by reactive oxygen species (ROS).

### Superoxide dismutase (SOD) activity

We measured SOD activity in transfected cell samples (NC1 and miR-335 inhibitor) at 24, 48 and 72 h, using the SOD colorimetric activity kit (Invitrogen) following the manufacturer’s instructions. We used curve-fitting software to generate standard curve because a four parameter algorithm provides the best standard curve fit (https://www.myassays.com).

### Caspase 3/7 assay

The caspase activity assay (Caspase-Glo 3/7 assay, Promega) was performed at 24, 48 and 72 h after transfection of SH-SY5Y cells with miR-335 inhibitor and following the manufacturer’s instructions.

### Western-Blot

WB was performed as previously described^46^. Protein extraction was performed with RIPA buffer (Sigma-Aldrich). 20 μg of protein was loaded in each well. Gels were transferred to nitrocellulose transfer membranes. Unspecific binding sites on the blots were blocked by incubation for 1 hour in Odyssey Blocking Buffer (LI-COR Biosciences, Lincoln, NE, USA) diluted 1:1 in phosphate buffer saline (PBS). The blots were incubated for 1 hour with the primary antibodies rabbit anti-caspase-3 (1/250 dilution) (Genetex, CA, USA, Cat#GTX110543, RRID:AB_10722709), mouse monoclonal anti-caspase-7 (1/250 dilution) (Thermo Fisher Scientific, Cat#MA1-91896, RRID:AB_2068163) and rabbit anti-p62 (1/250 dilution)(Enzo LifeSciences, Cat# BML-PW9860, RRID:AB_2196009). To normalize the results, mouse anti-β-actin (1/1000 dilution) (Sigma-Aldrich, Cat# A5441, RRID:AB_476744) was added simultaneously with the primary antibodies. The secondary antibodies used were goat anti-rabbit labeled with IR-Dye 680 (1/5000 dilution) (LI-COR Biosciences Cat# 926-32221, RRID:AB_621841) and goat anti-mouse labeled with IR-Dye 800 (1/5000 dilution) (LI-COR Biosciences Cat# 926-32210, RRID:AB_621842). After extensive washing, the immunoreactive bands were visualized and quantified using the Odyssey Infrared Imaging System (LI-COR).

### Statistical analysis

Experimental data were processed using SPSS Statistics 21.0 software (IBM Corp. Armonk, NY, USA). Normality and homogeneity of variance tests were performed. The data conforming to normal distribution and homogeneity of variance were expressed as mean ± standard deviation and analyzed with t-test^45^.

P values of <0.05 are indicated with one asterisk (*) and P values of less or equal than 0.01 are indicated with two asterisks (**).
